# Sphingosine 1‐phosphate attenuates neuronal dysfunction induced by amyloid‐β oligomers through endocytic internalization of NMDA receptors

**DOI:** 10.1111/febs.16579

**Published:** 2022-08-12

**Authors:** Alessandra Bigi, Roberta Cascella, Giulia Fani, Caterina Bernacchioni, Francesca Cencetti, Paola Bruni, Fabrizio Chiti, Chiara Donati, Cristina Cecchi

**Affiliations:** ^1^ Department of Experimental and Clinical Biomedical Sciences University of Florence Italy

**Keywords:** Alzheimer's disease, calcium dyshomeostasis, misfolded protein oligomers, sphingolipid, sphingosine 1‐phosphate receptors

## Abstract

Soluble oligomers arising from the aggregation of the amyloid beta peptide (Aβ) have been identified as the main pathogenic agents in Alzheimer's disease (AD). Prefibrillar oligomers of the 42‐residue form of Aβ (Aβ_42_O) show membrane‐binding capacity and trigger the disruption of Ca^2+^ homeostasis, a causative event in neuron degeneration. Since bioactive lipids have been recently proposed as potent protective agents against Aβ toxicity, we investigated the involvement of sphingosine 1‐phosphate (S1P) signalling pathway in Ca^2+^ homeostasis in living neurons exposed to Aβ_42_O. We show that both exogenous and endogenous S1P rescued neuronal Ca^2+^ dyshomeostasis induced by toxic Aβ_42_O in primary rat cortical neurons and human neuroblastoma SH‐SY5Y cells. Further analysis revealed a strong neuroprotective effect of S1P_1_ and S1P_4_ receptors, and to a lower extent of S1P_3_ and S1P_5_ receptors, which activate the G_i_‐dependent signalling pathways, thus resulting in the endocytic internalization of the extrasynaptic GluN2B‐containing *N*‐methyl‐*D*‐aspartate receptors (NMDARs). Notably, the S1P beneficial effect can be sustained over time by sphingosine kinase‐1 overexpression, thus counteracting the down‐regulation of the S1P signalling induced by Aβ_42_O. Our findings disclose underlying mechanisms of S1P neuronal protection against harmful Aβ_42_O, suggesting that S1P and its signalling axis can be considered promising targets for therapeutic approaches for AD.

## Graphical abstract

Both exogenous and endogenous sphingosine 1‐phosphate (S1P) rescue neuronal Ca^2+^ dyshomeostasis induced by prefibrillar oligomers of Aβ_42_ peptide (Aβ_42_O) in neuronal cells. S1P activates its specific receptors (S1PRs), which stimulate the endocytic internalization of the extrasynaptic GluN2B‐containing *N*‐methyl‐*D*‐aspartate receptors (NMDARs). Notably, the S1P beneficial effect can be sustained over time by sphingosine kinase‐1 (SK1) overexpression, thus counteracting the down‐regulation of the S1P signalling induced by Aβ_42_O.

Created with BioRender.com.

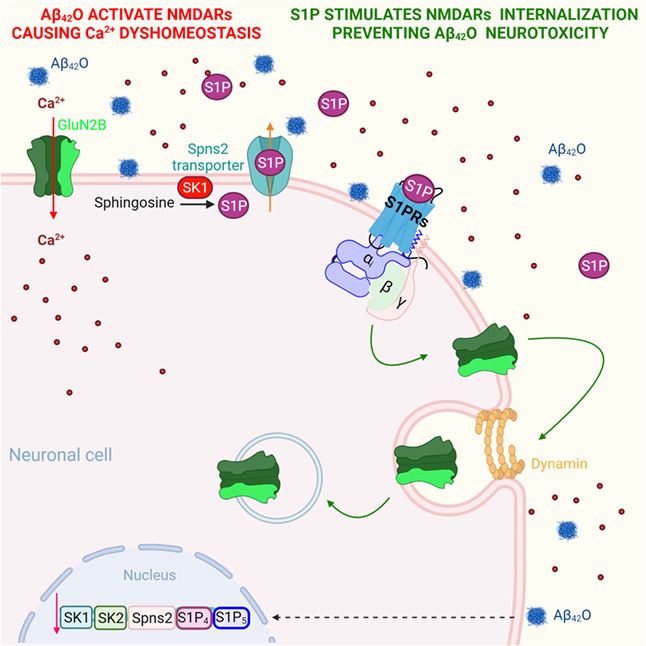

**Abbreviations**
AD: Alzheimer's disease
Aβ: amyloid beta peptide
Aβ_42_M: monomeric peptide
Aβ_42_O: Aβ oligomers
BDNF: brain‐derived neurotrophic factor
DMEM: Dulbecco's modified Eagle's medium
EMA: European Medicine Agency
FDA: American Food Drug Agency
HEPES: (2‐hydroxyethyl) piperazine‐1‐ethanesulfonic acid
mem: memantine
MTT: 3‐(4,5‐dimethylthiazol‐2‐yl)‐2,5 diphenyltetrazolium bromide
NMDARs: *N*‐methyl‐*D*‐aspartate receptors
PTX: Pertussis toxin
S1P: sphingosine 1‐phosphate
SK: sphingosine kinases
SPL: S1P lyase
Spns2: specific transporter spinster homologue 2
SPP1: S1P phosphatases 1

## Introduction

Alzheimer's disease (AD) is a devastating and fatal neurodegenerative condition affecting ca. 35 million people worldwide. The amyloid hypothesis proposes that the cardinal pathological feature of AD is the presence of senile plaques in the brain of affected people, predominantly formed by the misfolded and self‐assembled Aβ [1]. The aggregation process of Aβ is extremely complex and produces a large variety of oligomers, protofibrils and fibrils [2, 3]. Soluble Aβ_42_O formed early during Aβ aggregation, through secondary nucleation or released from mature fibrils, are considered to be the most potent Aβ neurotoxins implicated in AD [3, 4, 5].

Increasing evidence suggests that the dysregulation of cytosolic Ca^2+^ homeostasis is an upstream event evoked by Aβ_42_O in cultured neuronal cells and in relevant mouse AD models, where Ca^2+^ ions flow from the extracellular space to the cytosol [6, 7, 8, 9, 10, 11, 12]. In addition, the Ca^2+^ overload has been coupled to the deposition of senile plaques, as it appears to be most pronounced in the immediate vicinity of amyloid deposits in transgenic mouse models [13]. Furthermore, increased cytosolic Ca^2+^ levels can promote Aβ production and the microtubule‐associated protein tau phosphorylation, implicating calcium dysfunction as a possible causative factor in AD [8, 9].

The abnormal Ca^2+^ entry in cells arises from the Aβ_42_O ability to destabilize the cell membrane [8], as well as to activate a number of calcium channels, including the voltage‐gated calcium channels [14, 15] and receptor‐operated channels such as glutamatergic *N*‐methyl‐*D*‐aspartate receptors (NMDARs) [10, 16, 17, 18]. In particular, the abnormal activation of extrasynaptic NMDARs, predominantly composed of the GluN2B subunit, is considered a crucial mechanism in the Aβ_42_O‐induced neurotoxicity, responsible for synaptic dysfunction [19]. Consistently, the blockade of GluN2B‐containing NMDARs with specific antagonists, as well as the deletion of this subunit, were able to rescue Aβ‐induced neurotoxicity [20, 21]. In this regard, one of the drugs approved by the American Food Drug Agency (FDA), the European Medicine Agency (EMA), and other regulatory agencies is memantine (mem), which targets mainly extrasynaptic NMDARs as opposed to synaptic receptors [22].

Sphingosine 1‐phosphate (S1P) is a potent bioactive sphingolipid involved in the regulation of cellular Ca^2+^ signals along with differentiation, stress resistance, survival and regulation of neurotransmitter release in the central nervous system [23, 24]. S1P is synthetized by sphingosine phosphorylation, catalysed by sphingosine kinases (SK) 1 and 2 and is catabolized irreversibly by S1P lyase (SPL), or through the reversible dephosphorylation to sphingosine, catalysed by S1P phosphatases 1 (SPP1) and 2 (SPP2) [25, 26]. Although SK1 and SK2 catalyse the same reaction, their different intracellular localization, kinetic properties and tissue distribution are responsible in some cases for different, even opposite, biological outcomes [25]. S1P can function as an intracellular messenger, or be released in the extracellular space via the specific transporter spinster homologue 2 (Spns2) [27]. Once outside the cells, S1P acts as a ligand of five distinct G protein‐coupled receptors S1PR, referred to as S1P_1–5_ [28].

A significant reduction in S1P levels was evident in the hippocampus of elderly people, suggesting that its loss can predispose them to neurodegeneration [29]. Notably, a decreased level of S1P has also been observed in the brain of AD patients with a negative correlation with the amount of aggregated Aβ [30, 31]. Significant dysregulation of S1P_1_ expression, the most represented S1P receptor in the brain, was also reported to be associated with the defective S1P signalling evoked by Aβ accumulation [32]. Recently, an S1P analogue named fingolimod (FTY720), currently used in the therapy of multiple sclerosis, was observed to revert memory deficits in a rat model of AD, strongly suggesting a prominent role of S1P signalling in neuroprotection against Aβ toxicity [33]. Sub‐chronic treatment with fingolimod was also shown to decrease Ca^2+^ responsiveness of neurons to neurotoxic Aβ_42_O, thus ameliorating the cognitive performance in transgenic APPswe/PS1E9 AD mice [34].

In this study, we investigated the molecular mechanisms underlying the protective effects of S1P from Aβ_42_O neurotoxicity in primary rat cortical neurons and human SH‐SY5Y neuroblastoma cells. Our results indicate that S1P modulates NMDAR activation and the Ca^2+^ influx induced by Aβ_42_O. Indeed, S1P transiently reduces the surface localization of the extrasynaptic GluN2B subunit of NMDARs through its endocytic internalization, occurring upon S1P_1_ and S1P_4_, and to a lower extent S1P_3_ and S1P_5_, activation, thus eliciting beneficial effects. Moreover, we provide important evidence for a protective effect of SK1 overexpression against Aβ_42_O‐mediated damage in neuronal cells.

## Results

### S1P reduces mitochondrial dysfunction, apoptosis and Ca^2+^ dyshomeostasis induced by toxic Aβ_42_O

**Fig. 1:**
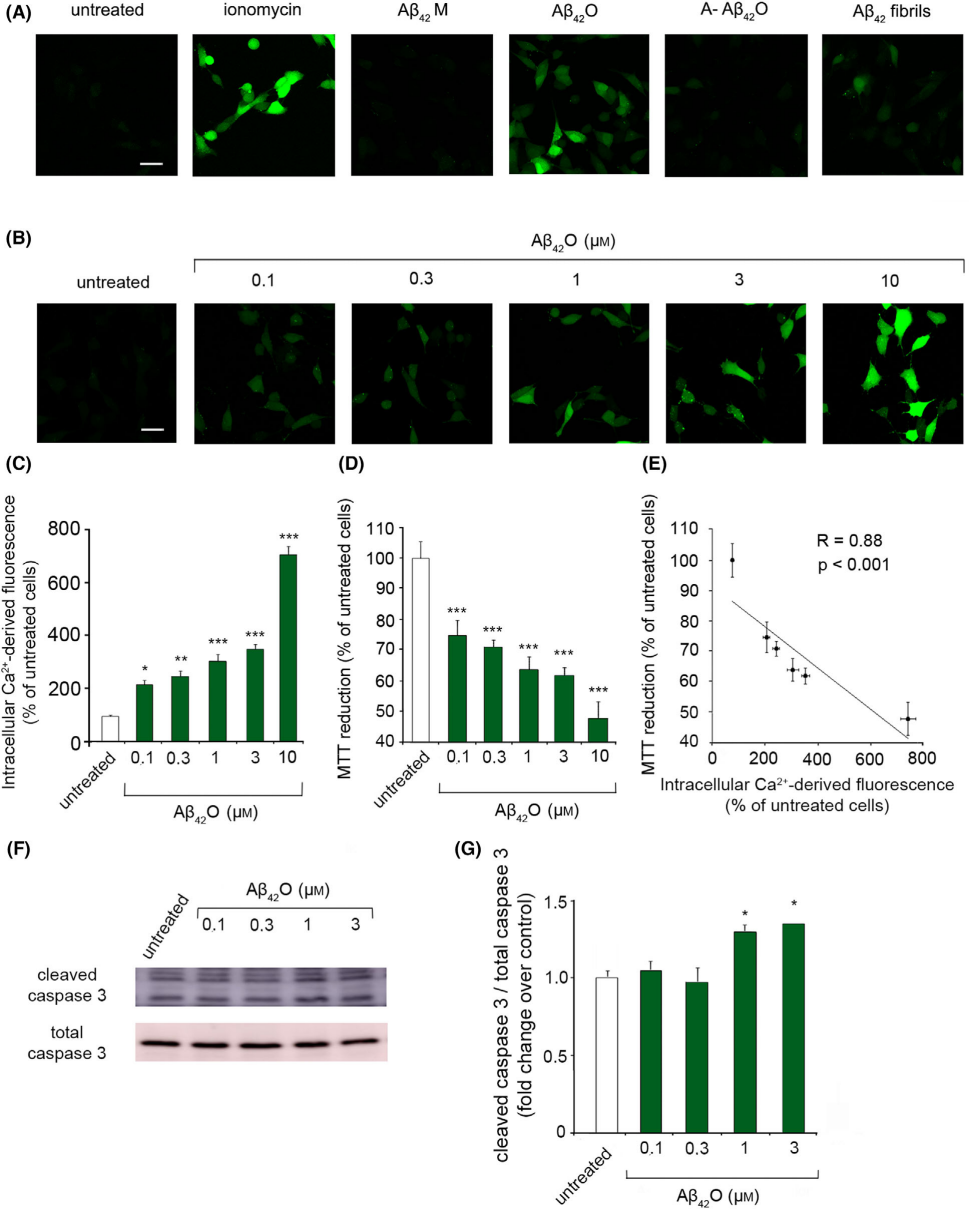
(A) Representative confocal microscope images showing the Ca^2+^‐derived fluorescence in SH‐SY5Y cells treated for 15 min with 1 μm ionomycin, or with the indicated Aβ_42_ species at 3 μm and then loaded with the Fluo‐4 AM probe. Untreated cells are also shown. (B) Representative confocal microscope images showing the Ca^2+^‐derived fluorescence in SH‐SY5Y cells treated for 15 min with increasing concentrations (0.1, 0.3, 1, 3 and 10 μm) of Aβ_42_O and then loaded with the Fluo‐4 AM probe. Untreated cells are also shown. (C) Semi‐quantitative analysis of the intracellular Ca^2+^‐derived fluorescence referring to panel (B). (D) MTT reduction in SH‐SY5Y cells treated for 24 h with increasing concentrations (0.1, 0.3, 1, 3 and 10 μm) of Aβ_42_O. (E) MTT reduction values reported in panel (D) plotted against the Ca^2+^‐derived fluorescence reported in panel (C) at the corresponding concentrations. In all panels, data are expressed as the percentage of the value for untreated cells. (F) Representative western blotting analysis of cell lysates from SH‐SY5Y cells treated for 24 h with increasing concentrations (0.1, 0.3, 1 and 3 μm) of Aβ_42_O. Caspase‐3 cleavage was measured in total cell lysate by using a specific anti‐caspase‐3 antibody. (G) Band intensity of western blotting in (F) was quantified by densitometric analysis and normalized to the expression of the total caspase‐3. Data are expressed as fold increase relative to the untreated sample, set as 1. Experimental errors are SEM (*n* = 3). A total of 60–100 cells (B, C), 200 000–250 000 cells (D) and 15 μg of cell lysates (F, G) were analysed per condition. Samples were analysed by one‐way ANOVA followed by Bonferroni's multiple‐comparison test relative to untreated cells (*P < 0.05, **P < 0.01, ***P < 0.001) in panels (C) and (D), or by one‐way ANOVA followed by Bonferroni's *post hoc* test relative to untreated cells (*P < 0.05) in panel (G). Scale bars, 30 μm.

In this work, we exploited highly stable and well‐characterized Aβ_42_ oligomers (Aβ_42_O) cross‐reacting with the conformation‐specific A11 antibody, referred to as prefibrillar oligomers [4] or A+ oligomers [35]. Previous evidence showed that these small and soluble aggregates cause massive neurological damage by impairing mitochondrial activity, inducing lactate dehydrogenase release and affecting Ca^2+^ homeostasis of neurons [4, 35, 36, 37]. Using dot blot assays and confocal microscopy coupled to immunofluorescence with A11 antisera, these species were also found in AD brain lysates [4], as well as in AD brain specimens analysed with immunogold electron microscopy [38, 39]. Here, we firstly compared the ability of Aβ_42_O to affect cytosolic Ca^2+^ homeostasis of human SH‐SY5Y neuroblastoma cells with respect to monomeric Aβ_42_, non‐toxic prefibrillar Aβ_42_ oligomers, that do not react with the conformation‐specific A11 antibody (A‐ Aβ_42_O) and Aβ_42_ fibrillar aggregates [35]. When Aβ_42_O were added to the culture medium for 15 min at a concentration of 3 μm (monomer equivalents), they caused an extensive influx of Ca^2+^ ions, whereas Aβ_42_ fibrils slightly increased the cellular Ca^2+^ level. On the contrary, A‐ Aβ_42_O and the monomeric peptide (Aβ_42_M) appeared to be innocuous (Fig. 1A). A dramatic increase in the Ca^2+^‐derived fluorescence was also observed in cells exposed to the ionophore ionomycin (Fig. 1A). Moreover, increasing concentrations (0.1, 0.3, 1, 3 and 10 μm, monomer equivalents) of Aβ_42_O triggered an overload of intracellular Ca^2+^ ions with a clear dose dependence up to 748 ± 27% employing 10 μm Aβ_42_O (Fig. 1B,C).

The neurotoxic effect of Aβ_42_O was also monitored by evaluating the reduction of 3‐(4,5‐dimethylthiazol‐2‐yl)‐2,5 diphenyltetrazolium bromide (MTT) [4, 40, 41]. When increased concentrations of Aβ_42_O were added to the culture medium of SH‐SY5Y cells for 24 h, the ability of cells to reduce MTT progressively decreased, reaching a value of 47 ± 5% at 10 μm Aβ_42_O with respect to untreated cells, taken as 100% (Fig. 1D). When the increase in the intracellular Ca^2+^ levels (Fig. 1C) was plotted versus the MTT reduction (Fig. 1D), a highly significant correlation between Ca^2+^ dyshomeostasis and Aβ_42_O‐induced mitochondrial dysfunction was found (*r* = 0.88, *P* < 0.001), (Fig. 1E). Consistently, 1 and 3 μm Aβ_42_O were found to cause apoptotic cell death, inducing caspase‐3 cleavage measured by western blot analysis (Fig. 1F,G).

**Fig. 2:**
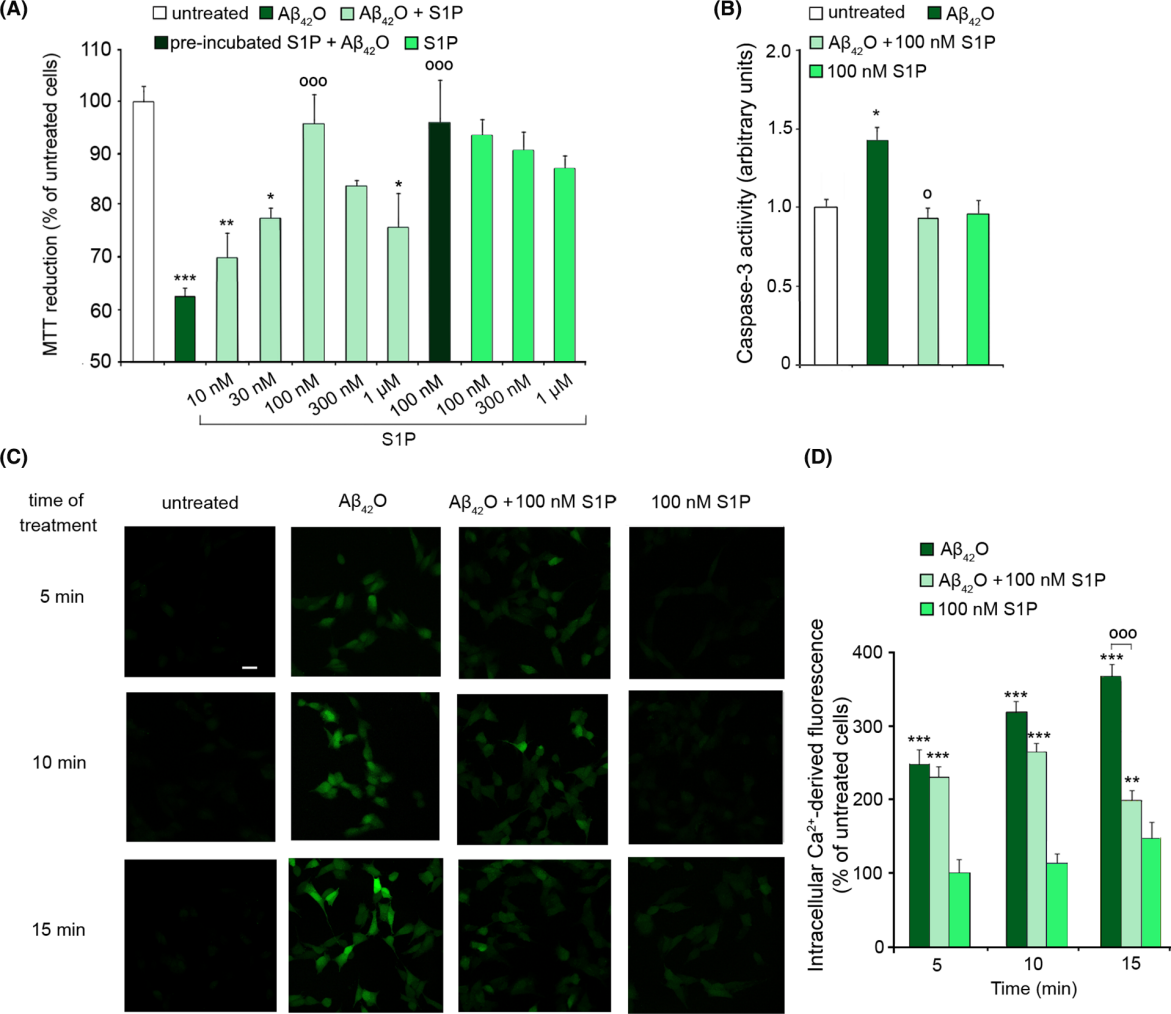
(A) MTT reduction in SH‐SY5Y cells treated for 24 h with 3 μm Aβ_42_O in the absence or presence of a co‐incubation with increasing concentrations (10, 30, 100, 300 nm and 1 μm) of S1P. Cells were also pre‐incubated with 100 nm S1P for 15 min and then treated with 3 μm Aβ_42_O. S1P alone at increasing concentrations (100, 300 nm and 1 μm) was also tested as a control. Data are expressed as the percentage of the value for untreated cells. (B) Fluorimetric analysis of the caspase‐3 activity in SH‐SY5Y treated for 24 h with 3 μm Aβ_42_O in the absence or presence of 100 nm S1P. Cells were also treated with 100 nm S1P alone as a control. Data are expressed as fold increase relative to the untreated sample, set as 1. (C) Representative confocal microscope images showing the Ca^2+^‐derived fluorescence in SH‐SY5Y cells treated for 5, 10 and 15 min with 3 μm Aβ_42_O in the absence or presence of 100 nm S1P and then loaded with the Fluo‐4 AM probe. Untreated cells and cells treated with 100 nm S1P alone are also shown. (D) Semi‐quantitative analysis of the intracellular Ca^2+^‐derived fluorescence referring to panel (C). Data are expressed as the percentage of the value for untreated cells. Experimental errors are SEM (*n* = 4 in panel (A) and *n* = 3 in panels (B–D). A total of 200 000–250 000 cells (A), 15 μg of cell lysates (B) and 60–100 cells (C, D) were analysed per condition. Samples were analysed by one‐way ANOVA followed by Bonferroni's multiple‐comparison test relative to untreated cells (*P < 0.05, **P < 0.01, ***P < 0.001) and to cells treated with Aβ_42_O (°°°*P* < 0.001) in panels (A) and (D), or by Student's *t* test relative to untreated cells (*P < 0.05) and to cells treated with Aβ_42_O in the absence of S1P (°*P* < 0.05), in panel (B). Scale bar, 30 μm.

We then examined whether S1P could inhibit Aβ_42_O neurotoxicity in cultured cells. We first monitored the mitochondrial dysfunction by treating SH‐SY5Y cells for 24 h with 3 μm Aβ_42_O in the absence or presence of S1P at concentrations ranging from 10 nm to 1 μm. The MTT reduction was significantly restored in the presence of 100 nm S1P, either pre‐incubated or co‐incubated with the oligomers (Fig. 2A), with a minor protective effect observed at both lower and higher concentrations, showing a bell‐shaped S1P dependence (Fig. 2A). This reduced protection cannot be ascribed to inherent neurotoxicity of S1P *per se*, since the addition of 1 μm S1P to the culture medium in the absence of Aβ_42_O was not cytotoxic to SH‐SY5Y cells (Fig. 2A). Accordingly, the activation of caspase‐3 triggered by 3 μm Aβ_42_O added to the cell culture medium for 24 h was completely prevented by 100 nm S1P co‐incubated with the oligomers, demonstrating a protective role of this sphingolipid against apoptotic cell death (Fig. 2B). As control, cells were also treated with S1P in the absence of Aβ_42_O, without detecting any cytotoxic effect at the tested concentrations (Fig. 2B). On the basis of these results, 100 nm S1P was employed in all subsequent experiments.

We then investigated whether S1P was able to rescue the early Ca^2+^ dyshomeostasis induced by Aβ_42_O in SH‐SY5Y cells. Thus, cells were treated with 3 μm Aβ_42_O for different lengths of time (5, 10 and 15 min), in the absence or presence of 100 nm S1P. Confocal microscopy analysis revealed that Aβ_42_O evoked a rapid and significant increase in the intracellular Ca^2+^ levels already after 5 min of treatment (by 250 ± 19% relative to untreated cells, taken as 100%), reaching higher levels at 10 and 15 min of treatment (by 265 ± 10% and 366 ± 15%, respectively) (Fig. 2C,D). The protective effect of S1P was significant at 15 min of treatment, with a reduction in Ca^2+^ dyshomeostasis by 167 ± 25% (Fig. 2C,D). Cells exposed to 100 nm S1P in the absence of Aβ_42_O for 15 min also showed a minor Ca^2+^ increase, in good agreement with the observed role of S1P in Ca^2+^ signals [42, 43].

### S1PR mediates the protective effect of S1P against Aβ_42_O neurotoxicity

**Fig. 3:**
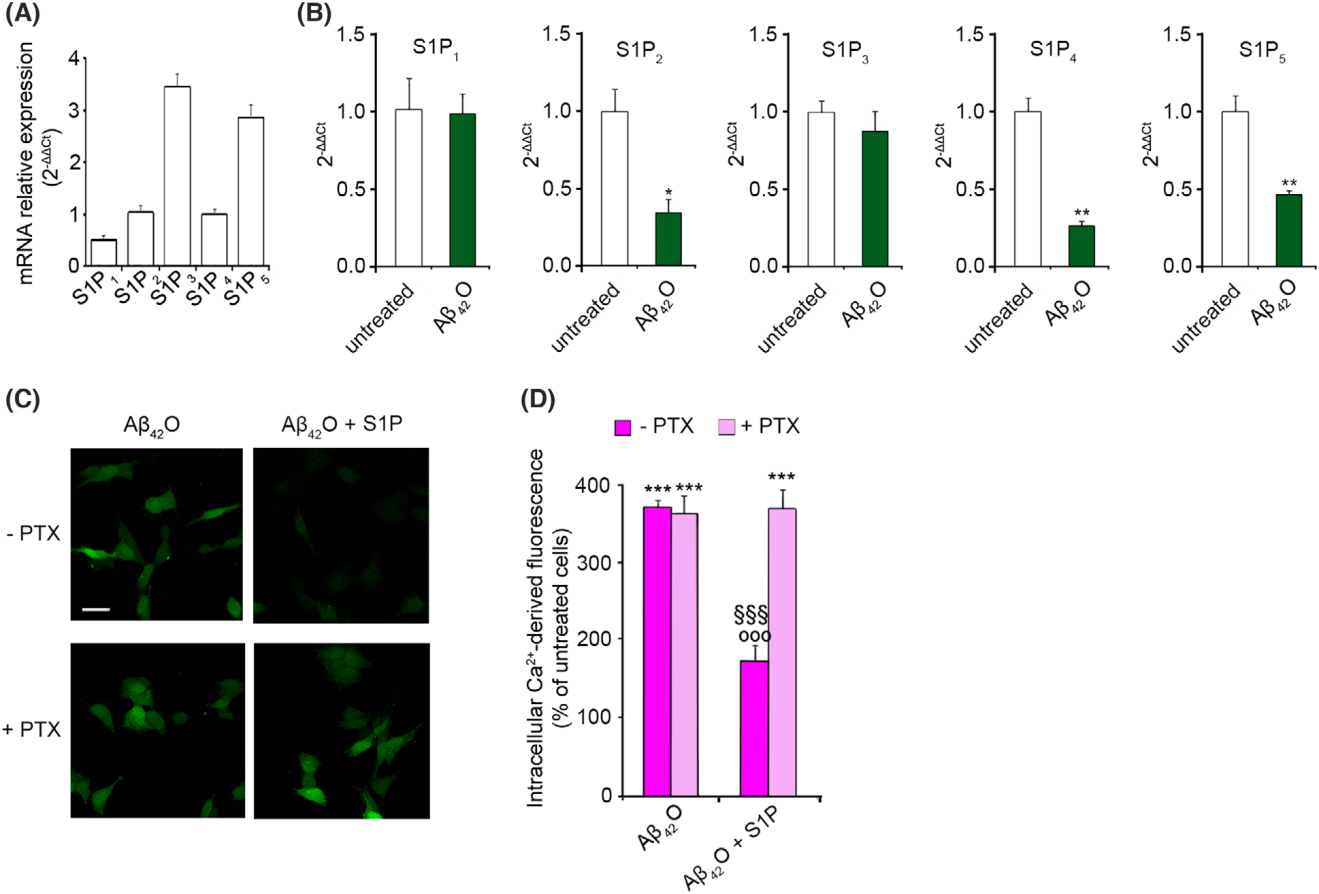
(A) Quantitative mRNA analysis of S1P_1–5_ in SH‐SY5Y cells. Data are expressed according to the $2^{-\Delta \Delta Ct}$ method, using S1P_4_ as calibrator. (B) Quantitative mRNA analysis of S1P_1–5_ in SH‐SY5Y cells treated for 24 h with 3 μm Aβ_42_O. S1PR mRNA quantitation was based on the $2^{‐\Delta \Delta Ct}$ method, using individual S1PR subtype of the unchallenged specimen as calibrator. (C) Representative confocal microscope images showing the Ca^2+^‐derived fluorescence in SH‐SY5Y cells treated for 15 min with 3 μm Aβ_42_O in the absence or presence of 100 nm S1P, following 4 h of pre‐treatment with 200 ng·mL^−1^ PTX. Cells were then loaded with the Fluo‐4 AM probe. (D) Semi‐quantitative analysis of the intracellular Ca^2+^‐derived fluorescence referring to panel (C). Data are expressed as the percentage of the value for untreated cells. Experimental errors are SEM (*n* = 3). A total of 1–2 μg of RNA (A, B) and 60–100 cells (C, D) were analysed per condition. Samples were analysed by Student's *t* test relative to untreated cells (*P < 0.05, **P < 0.01) in panel (B), or by one‐way ANOVA followed by Bonferroni's multiple‐comparison test relative to untreated cells (***P < 0.001), to cells treated with Aβ_42_O in the absence of S1P and PTX (°°°*P* < 0.001) and to cells treated with Aβ_42_O + S1P in the presence of PTX (^§§§^ *P* < 0.001) in panel (D). Scale bar, 30 μm.

We then examined whether the protective effect of S1P against Aβ_42_O toxicity was mediated by S1PR. Real‐time PCR analysis of the mRNA expression levels of the S1PR family showed that all S1PRs were expressed in SH‐SY5Y cells, with S1P_3_ appearing to be the most abundant (Fig. 3A). In spite of their relative abundance, the treatment of cells with 3 μm Aβ_42_O for 24 h caused a significant down‐regulation of the mRNA expression of S1P_2_, S1P_4_ and S1P_5_, whereas S1P_1_ and S1P_3_ levels did not change (Fig. 3B).

Since all S1PRs, even not exclusively, couple with G_i_ proteins [44], we pre‐treated the cells for 4 h with 200 ng·mL^−1^ Pertussis toxin (PTX), a specific inhibitor of G_i_/G_o_ proteins [45], to investigate the involvement of S1PR in mediating the S1P neuroprotective effect in SH‐SY5Y cells. The beneficial effect of S1P on Aβ_42_O‐induced Ca^2+^ entry was completely lost in the presence of PTX (Fig. 3C,D), demonstrating that the S1P action was G_i_ dependent.

**Fig. 4:**
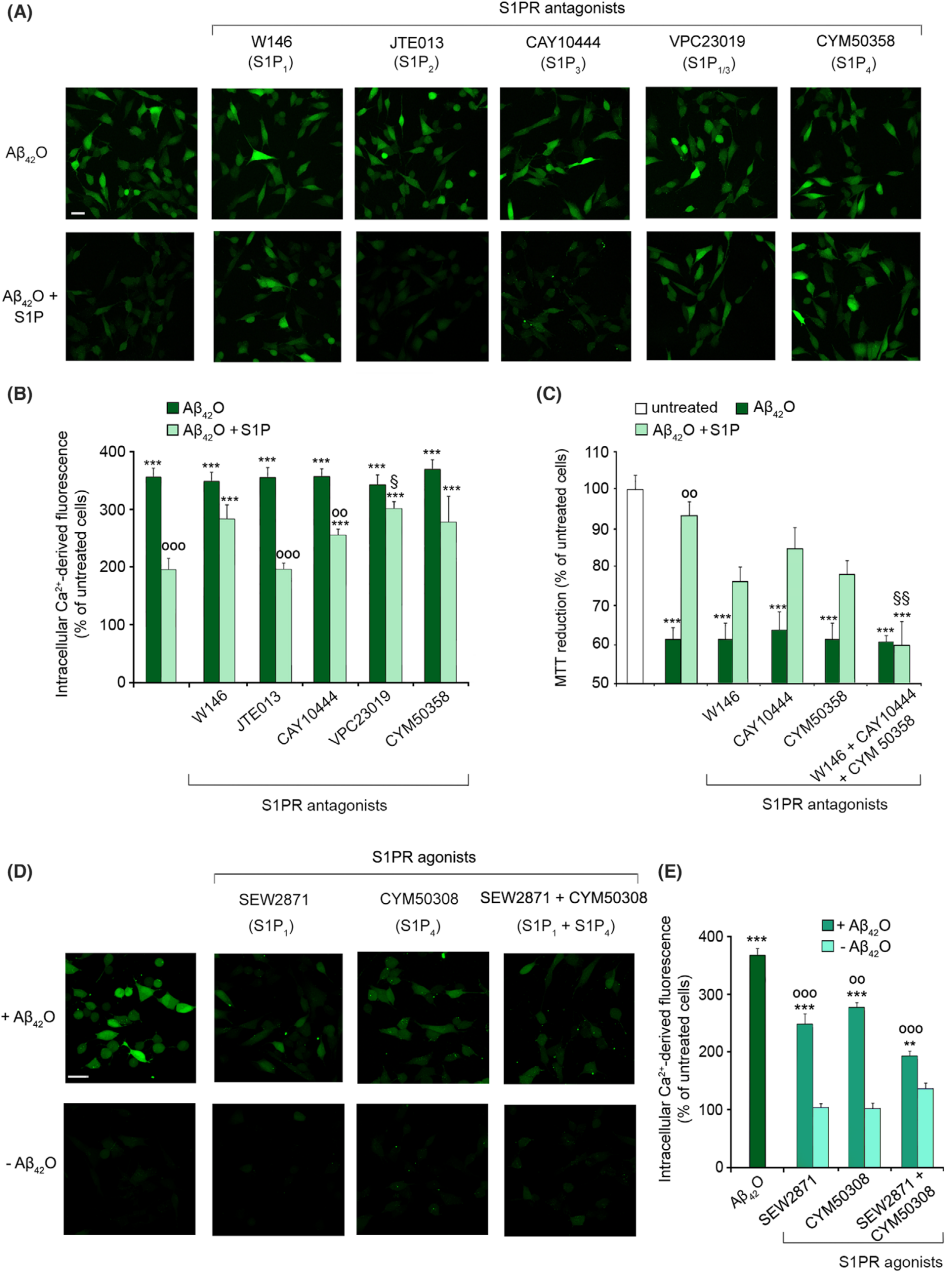
(A) Representative confocal microscope images showing the Ca^2+^‐derived fluorescence in SH‐SY5Y cells treated for 15 min with 3 μm Aβ_42_O in the absence or presence of 100 nm S1P, following 30 min of pre‐treatment with the S1PR antagonists W146 (S1P_1_) at 10 μm, JTE013 (S1P_2_) at 1 μm, CAY10444 (S1P_3_) at 5 μm, VPC23019 (S1P_1_ and S1P_3_) at 1 μm and CYM50358 (S1P_4_) at 1 μm. Cells were then loaded with the Fluo‐4 AM probe. (B) Semi‐quantitative analysis of the intracellular Ca^2+^‐derived fluorescence referring to panel (A). (C) MTT reduction in SH‐SY5Y cells treated for 24h with 3 μm Aβ_42_O in the absence or presence of 100 nm S1P, following 30 min of pre‐treatment with W146 at 10 μm, CAY10444 at 5 μm, CYM50358 at 1 μm and with all the antagonists together at the previously reported concentrations. (D) Representative confocal microscope images showing the Ca^2+^‐derived fluorescence in SH‐SY5Y cells treated for 15 min with 3 μm Aβ_42_O in the absence or presence of 100 nm S1P or the S1PR agonists SEW2871 (S1P_1_) at 10 μm and CYM50308 (S1P_4_) at 1 μm, and both agonists together at the previously reported concentrations. Cells were then loaded with the Fluo‐4 AM probe. (E) Semi‐quantitative analysis of the intracellular Ca^2+^‐derived fluorescence referring to panel (D). In all panels, data are expressed as the percentage of the value for untreated cells. A total of 80–120 cells (A–B, D–E) and 200 000–250 000 cells (C) were analysed per condition. Experimental errors are SEM (*n* = 3). Samples were analysed by one‐way ANOVA followed by Bonferroni's multiple‐comparison test relative to untreated cells (**P < 0.01, ***P < 0.001), to cells treated with Aβ_42_O alone (°°*P* < 0.01, °°°*P* < 0.001), and to cells treated with Aβ_42_O + S1P in the absence of S1PR antagonists (^§^ *P* < 0.05, ^§§^ *P* < 0.01). Scale bars, 30 μm.

Thus, the involvement of S1PR in mediating the protective effect of S1P against Aβ_42_O toxicity was evaluated with specific S1PR antagonists. To this aim, SH‐SY5Y cells were pre‐incubated with specific antagonists of S1P_1_, S1P_2_, S1P_3_ or S1P_4_ for 30 min, and then treated with 3 μm Aβ_42_O for 15 min, in the absence or presence of 100 nm S1P. The ability of S1P to reduce Aβ_42_O‐induced Ca^2+^ influx was partially impaired in the presence of the S1P_1_ or S1P_4_ selective antagonists W146 [46] and CYM50358 [47] respectively (light green bars in Fig. 4A,B). The inhibition of S1P_3_ by its specific antagonist CAY10444 partially reduced S1P protection, whereas that of of S1P_2_ by its specific antagonist JTE013 did not produce any detectable effect (Fig. 4A,B). The functional blockade of both S1P_1_ and S1P_3_ with the antagonist VPC23019 confirmed the role of such receptors in S1P‐mediated neuroprotection (Fig. 4A,B). S1PR antagonists did not affect the Ca^2+^ influx triggered by 3 μm Aβ_42_O in the absence of 100 nm S1P (Fig. 4A,B). Accordingly, the MTT test revealed that S1P protection was markedly reduced by the blockade of S1P_1_ and partially prevented by S1P_3_ and S1P_4_ antagonists, but fully reduced by the combined antagonism of all three receptors (Fig. 4C). Most studies that validate the S1PR‐blocking effects of S1P antagonists used concentrations equal to or lower than 1 μm, while evidence of non‐specificity at higher concentrations was found [48]. Thus, a slight inhibition of all S1PRs by a selective S1P antagonist cannot be ruled out in our experimental conditions. In addition, a cross‐talk between different S1PR signalling pathways could cause a minor response of other S1PR when a single S1PR was inhibited.

We next exploited the potent and high‐selective S1P_1_ and S1P_4_ agonists SEW2871 and CYM50308, respectively, to further confirm the role of these receptors in S1P axis [49, 50].

Thus, Aβ_42_O were added to the SH‐SY5Y cell culture medium at 3 μm for 15 min in the presence or absence of S1PR agonists at 1 μm. We observed a partial and complete reduction in the early Ca^2+^ dyshomeostasis evoked by Aβ_42_O when the two agonists were used separately and together, respectively (Fig. 4D,E).

**Fig. 5:**
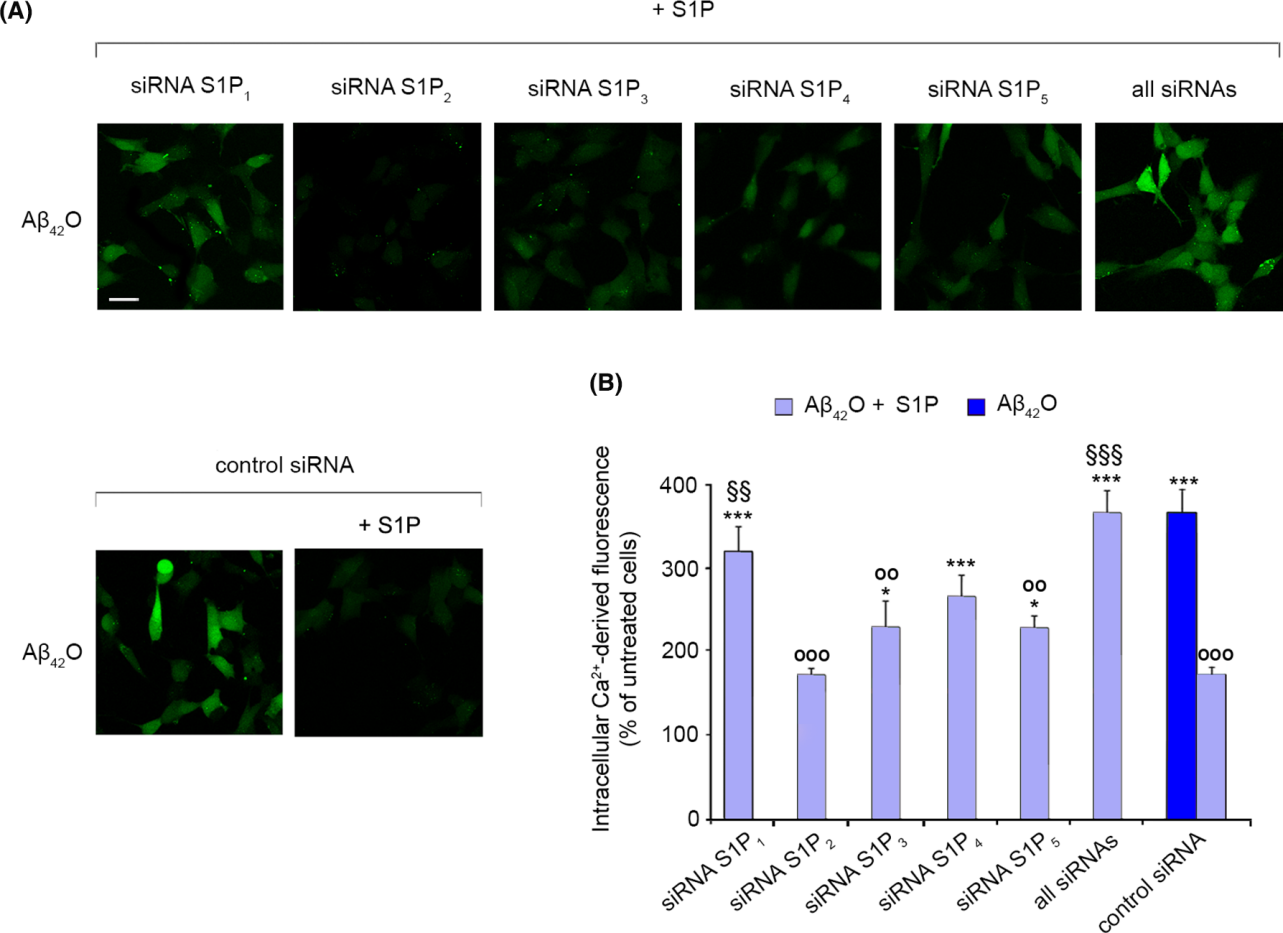
(A) Representative confocal microscope images showing the Ca^2+^‐derived fluorescence in SH‐SY5Y cells pre‐transfected with siRNA against S1P_1_, S1P_2_, S1P_3_, S1P_4_ and S1P_5_ receptors and with all the analysed siRNAs or control siRNA, and then treated for 15 min with 3 μm Aβ_42_O in the absence or presence of 100 nm S1P. Cells were then loaded with the Fluo‐4 AM probe. (B) Semi‐quantitative analysis of the intracellular Ca^2+^‐derived fluorescence referring to panel (A). Data are expressed as the percentage of the value for untreated cells. Experimental errors are SEM (*n* = 3). A total of 80–100 cells were analysed per condition. Samples were analysed by one‐way ANOVA followed by Bonferroni's multiple‐comparison test relative to untreated cells pre‐transfected with control siRNA (*P < 0.05, ***P < 0.001), to cells pre‐transfected with control siRNA and treated with Aβ_42_O alone (°°*P* < 0.01, °°°*P* < 0.001), and to cells pre‐transfected with control siRNA and then treated with Aβ_42_O + S1P (^§§^ *P* < 0.01, ^§§§^ *P* < 0.001). Scale bar, 30 μm.

To validate our results with a different method, the specific involvement of individual S1PR in the protective effect of S1P against Aβ_42_O toxicity was evaluated by RNA interference. Indeed, SH‐SY5Y cells were transfected with a siRNA‐negative control or with siRNAs against S1P_1_, S1P_2_, S1P_3_, S1P_4_ and S1P_5_, and then treated for 15 min with 3 μm Aβ_42_O in the absence or presence of 100 nm S1P. Aβ_42_O evoked a significant Ca^2+^ entry in cells transfected with the control siRNA (370 ± 27% relative to control cells), which was significantly reduced by 100 nm S1P (by 195 ± 36%). Notably, the S1P‐mediated reduction was remarkably lower in cells pre‐treated with siRNAs. The most effective was the siRNA against S1P_1_, followed by the siRNA against S1P_4_, and then S1P_3_ and S1P_5_, whereas the silencing of S1P_2_ did not impair the beneficial effect of S1P (Fig. 5A,B). Accordingly, S1P beneficial action was completely lost in cells transfected with a combination of all siRNAs (Fig. 5A,B). Overall, these data indicate that S1P protection against harmful Aβ_42_O involves S1P_1_, S1P_3_, S1P_4_ and S1P_5_.

### S1P rescues Ca^2+^ dyshomeostasis by modulating NMDARs exposure on neuronal membrane

**Fig. 6:**
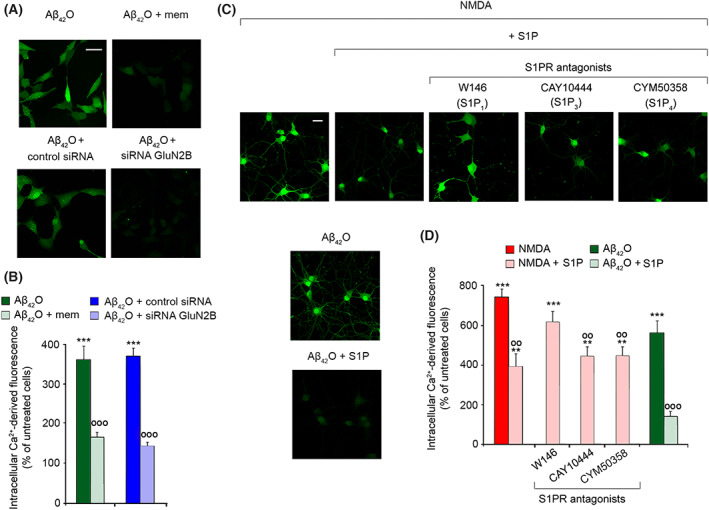
(A) Representative confocal microscope images showing the Ca^2+^‐derived fluorescence in SH‐SY5Y cells treated for 15 min with 3 μm Aβ_42_O in the absence or presence of a 60 min pre‐treatment with 10 μm mem (upper panels), or in the presence of a pre‐transfection with control siRNA and with siRNA against GluN2B subunit (lower panels). Cells were then loaded with the Fluo‐4 AM probe. (B) Semi‐quantitative analysis of the intracellular Ca^2+^‐derived fluorescence referring to panel (A). Data are expressed as the percentage of the value for untreated not‐tranfected cells, or untreated cells pre‐transfected with control siRNA. (C) Representative confocal microscope images showing the Ca^2+^‐derived fluorescence in primary rat cortical neurons treated with 1 mm NMDA in the absence or presence of 100 nm S1P, following 30 min of pre‐treatment with the S1PR antagonists W146 (S1P_1_) at 10 μm, CAY10444 (S1P_3_) at 5 μm and CYM50358 (S1P_4_) at 1 μm. Cells were also treated with 3 μm Aβ_42_O in the absence or presence of 100 nm S1P, and then loaded with the Fluo‐4 AM probe. (D) Semi‐quantitative analysis of the intracellular Ca^2+^‐derived fluorescence referring to panel (C). Data are expressed as the percentage of the value for untreated cells. Experimental errors are SEM (*n* = 3). A total of 60–100 cells (A, B) and 80–120 cells (C, D) were analysed per condition. Samples were analysed by one‐way ANOVA followed by Bonferroni's multiple‐comparison test relative to untreated cells (**P < 0.01, ***P < 0.001), to cells treated with Aβ_42_O in the absence of mem, or in the presence of the control siRNA (°°°*P* < 0.001) in panel (B), and to cells treated with NMDA or Aβ_42_O alone (°°*P* < 0.01, °°°*P* < 0.001) in panel (D). Scale bars, 30 μm (A) and 20 μm (C).

An increasing body of evidence strongly indicates that Aβ_42_O evoke a massive Ca^2+^ influx through the activation of NMDARs, particularly at the early stages of the observed Ca^2+^ uptake [12, 17, 18, 51]. We, therefore, assessed whether S1P prevents neuronal Ca^2+^ responsiveness by modulating NMDAR activity. Pre‐treatment of SH‐SY5Y cells with 10 μm mem, a low‐affinity NMDAR antagonist, significantly reduced the rapid Ca^2+^ increase evoked by 3 μm Aβ_42_O for 15 min (by 196 ± 48%), confirming a crucial role for NMDARs in the early neuronal Ca^2+^ dysregulation (Fig. 6A,B). Similarly, the silencing of the 2B subunit of NMDARs (GluN2B) by its specific siRNA in SH‐SY5Y cells also resulted in a significant reduction in intracellular Ca^2+^ levels (by 196 ± 31%) induced by 3 μm Aβ_42_O for 15 min, compared with cells transfected with a negative control siRNA and then treated with the same species (Fig. 6A,B).

When NMDARs were activated in primary rat cortical neurons with their specific agonist NMDA at 1 mm for 15 min, we observed a dramatic increase in intracellular calcium (by 745 ± 46%) with respect to untreated cells, which was slightly higher than that observed upon treatment with 3 μm Aβ_42_O (566 ± 66%) (Fig. 6C,D). Notably, the presence of 100 nm S1P significantly reduced the Ca^2+^ influx induced by both NMDA (by 356 ± 111%) and Aβ_42_O (by 423 ± 72%) respectively (Fig. 6C,D). The presence of the S1P_1_ antagonist W146 and, to a lesser extent, S1P_3_ and S1P_4_ antagonists CYM50358 and CAY10444, partially prevented the beneficial effect of S1P (Fig. 6C,D). These results indicate that S1P signalling helps the maintenance of Ca^2+^ homeostasis in NMDA‐stimulated primary rat cortical neurons by blocking the Ca^2+^ influx occurring via NMDARs (Fig. 6A–D).

**Fig. 7:**
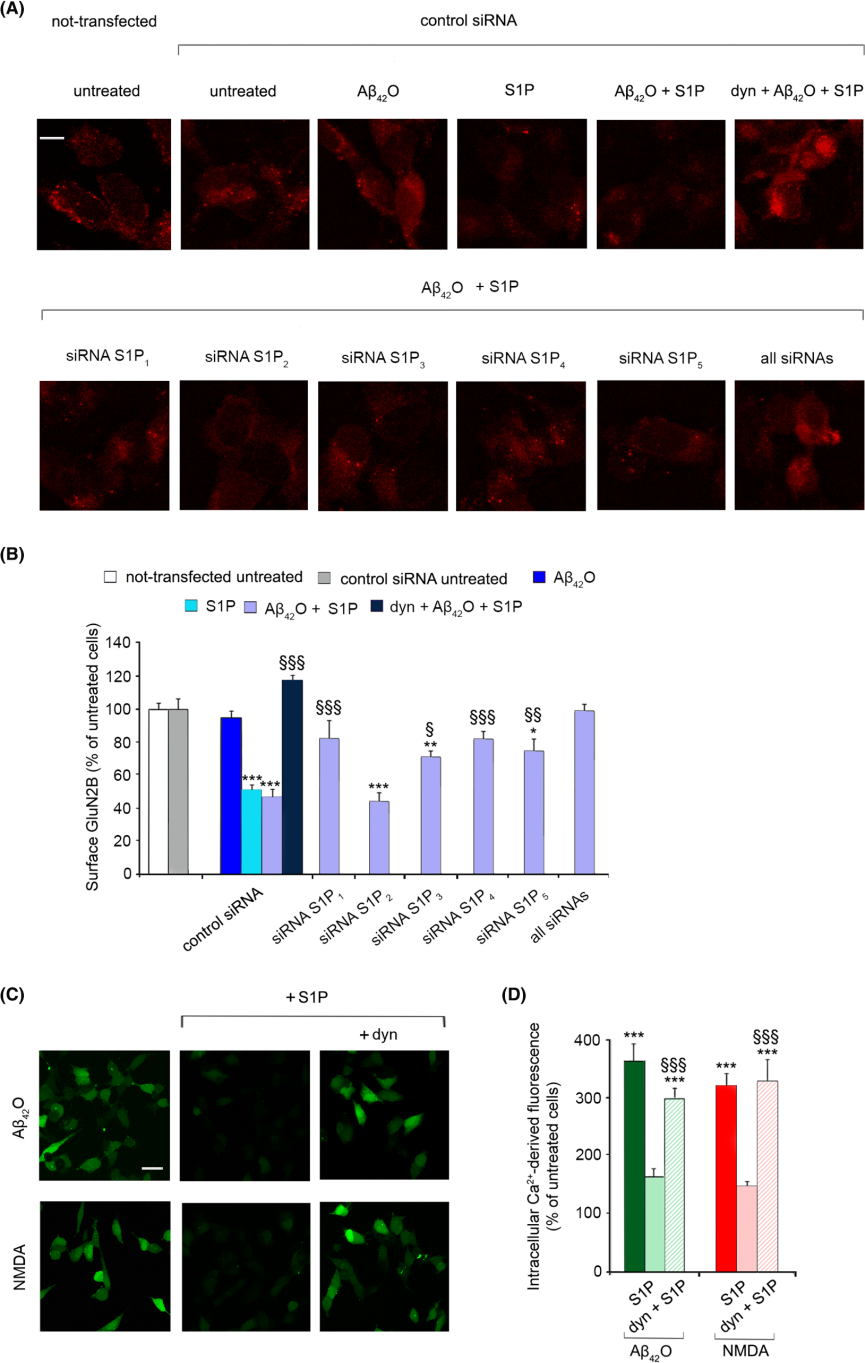
(A) Representative confocal microscope images showing the immunostaining of GluN2B subunit of NMDARs in SH‐SY5Y cells that were either not‐transfected, pre‐transfected with control siRNA, or pre‐transfected with siRNA against S1P_1_, S1P_2_, S1P_3_, S1P_4_ and S1P_5_ receptors and with all the five siRNAs, and then treated with 3 μm Aβ_42_O for 15 min in the absence or presence of 100 nm S1P, following 30 min of pre‐treatment with 50 μm dyn. (B) Semi‐quantitative analysis of the number of fluorescent puncta on the membrane surface of SH‐SY5Y cells referring to panel (A). Data are expressed as the percentage of the value for untreated cells pre‐transfected with control siRNA. (C) Representative confocal microscope images showing the Ca^2+^‐derived fluorescence in SH‐SY5Y cells treated for 15 min with 3 μm Aβ_42_O , or with 1 mm NMDA, in the absence or presence of 100 nm S1P, and following 30 min of pre‐treatment with 50 μm dyn. Cells were then loaded with the Fluo‐4 AM probe. (D) Semi‐quantitative analysis of the intracellular Ca^2+^‐derived fluorescence referring to panel (C). Data are expressed as the percentage of the value for untreated cells. Experimental errors are SEM (*n* = 3). A total of 80–120 cells (A, B) and 60–100 cells (C, D) were analysed per condition. Samples were analysed by one‐way ANOVA followed by Bonferroni's multiple‐comparison test relative to untreated cells (*P < 0.05, **P < 0.01, ***P < 0.001), to cells transfected with control siRNA and then treated with Aβ_42_O + S1P (^§^ *P* < 0.05, ^§§^ *P* < 0.01, ^§§§^ *P* < 0.001) in panel (B), and to cells treated with Aβ_42_O + S1P, or NMDA + S1P in the absence of dyn (^§§§^ *P* < 0.001) in panel (D). Scale bars, 10 μm (A) and 30 μm (C).

Then, we investigated whether the protective effect of S1P against Aβ_42_O could be mediated by relocation of extrasynaptic GluN2B‐containing NMDARs [51, 52]. Thus, the distribution of the NMDAR subunit GluN2B on the neuronal membrane of SH‐SY5Y cells was determined after transfecting cells with the control siRNA or with siRNA against the S1P receptors and then adding Aβ_42_O to the culture medium, in the absence or presence of S1P (Fig. 7A,B). We observed that 100 nm S1P and 100 nm S1P with 3 μm Aβ_42_O significantly reduced the membrane exposure of GluN2B (by 49 ± 9% and 53 ± 8%, respectively) in cells transfected with control siRNA, whereas 3 μm Aβ_42_O did not evoke any appreciable mobilization of the subunit (Fig. 7A,B). Consistently, the pre‐treatment of SH‐SY5Y cells with 50 μm dynasore (dyn), a cell‐permeable‐specific inhibitor of dynamin, totally prevented the clathrin‐mediated endocytosis of NMDARs (Fig. 7A,B). In such conditions, an overall inhibition of the physiological recycling of the NMDARs should be speculated, as the percentage of exposed GluN2B was slightly higher with respect to untreated cells. Notably, a reduced internalization of GluN2B was observed in cells pre‐treated with siRNA against S1P_1_, S1P_4_ and, to a minor extent, S1P_3_ and S1P_5_, whereas the silencing of S1P_2_ did not reduce the effect of S1P (Fig. 7A,B). Consistently, the simultaneous silencing of all S1PRs completely abolished GluN2B internalization (Fig. 7A,B).

According to data obtained in primary rat cortical neurons, NMDA induced a significant increase in intracellular calcium (by 320 ± 18% relative to untreated cells) in SH‐SY5Y cells, comparable to that observed when cells were treated with 3 μm Aβ_42_O (by 362 ± 37%) (Fig. 7C,D). The incubation with 100 nm S1P significantly reduced the Ca^2+^ influx evoked by both NMDA and Aβ_42_O (Fig. 7C,D), strengthening S1P role in the membrane depletion of extrasynaptic GluN2B‐containing NMDARs.

Notably, 50 μm dyn completely abolished the protective effect of S1P against the massive increase in intracellular Ca^2+^ evoked by 3 μm Aβ_42_O or 1 mm NMDA (Fig. 7C,D). Taken together, these results indicate that the early Ca^2+^ dysregulation induced by Aβ_42_O is mitigated by S1P through the dynamin‐dependent endocytosis of GluN2B‐containing NMDARs, occurring upon the activation of S1P receptors.

### Endogenous S1P protects against harmful Aβ_42_O

**Fig. 8:**
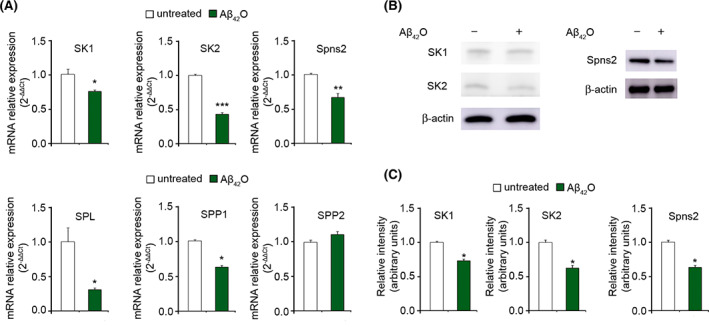
(A) Quantitative mRNA analysis of SK1, SK2, Spns2, SPL, SPP1 and SPP2 performed by a real‐time polymerase chain reaction in total RNA extracted from SH‐SY5Y cells treated with 3 μm Aβ_42_O for 24 h. S1P metabolism enzymes and Spns2 mRNA quantitation was based on the $2^{‐\Delta \Delta Ct}$ method, using individual enzyme or Spns2 of the unchallenged specimen as calibrator. (B) Western blotting analysis of SK1, SK2 and Spns2 expression in SH‐SY5Y cells treated with 3 μm. Aβ_42_O for 24 h. A blot representative of three independent experiments is shown. (C) Band intensity of western blotting shown in panel (B), quantified by densitometric analysis and normalized to the expression of β‐actin. Data are expressed as fold increase relative to the untreated specimen, set as 1. Experimental errors are SEM (*n* = 3). A total of 1–2 μg of RNA (A) and 15 μg of cell lysates (B, C) were analysed per condition. Samples were analysed by Student's *t* test relative to untreated cells (*P < 0.05, **P < 0.01, ***P < 0.001).

The ability of Aβ_42_O to modulate the mRNA expression levels of SK1, SK2 and Spns2 involved in S1P signalling axis was also evaluated in SH‐SY5Y cells exposed to 3 μm Aβ_42_O for 24 h. A significant down‐regulation of SK1 and SK2 expression was found, indicating an impairment of the S1P signalling evoked by Aβ_42_O (Fig. 8A). Aβ_42_O also induced a significant decrease in Spns2 expression (Fig. 8A). The mRNA expression levels of SPL and SPP1 were diminished, whereas those of SPP2 were not significantly altered by Aβ_42_O (Fig. 8A). The reduction in SK1, SK2 and Spns2 expression elicited by Aβ_42_O was also confirmed at the protein level by western blotting analysis (Fig. 8B,C). Thus, in line with literature data, the Aβ_42_O toxic effect envisages the alteration of the metabolism and signalling of S1P [53].

**Fig. 9:**
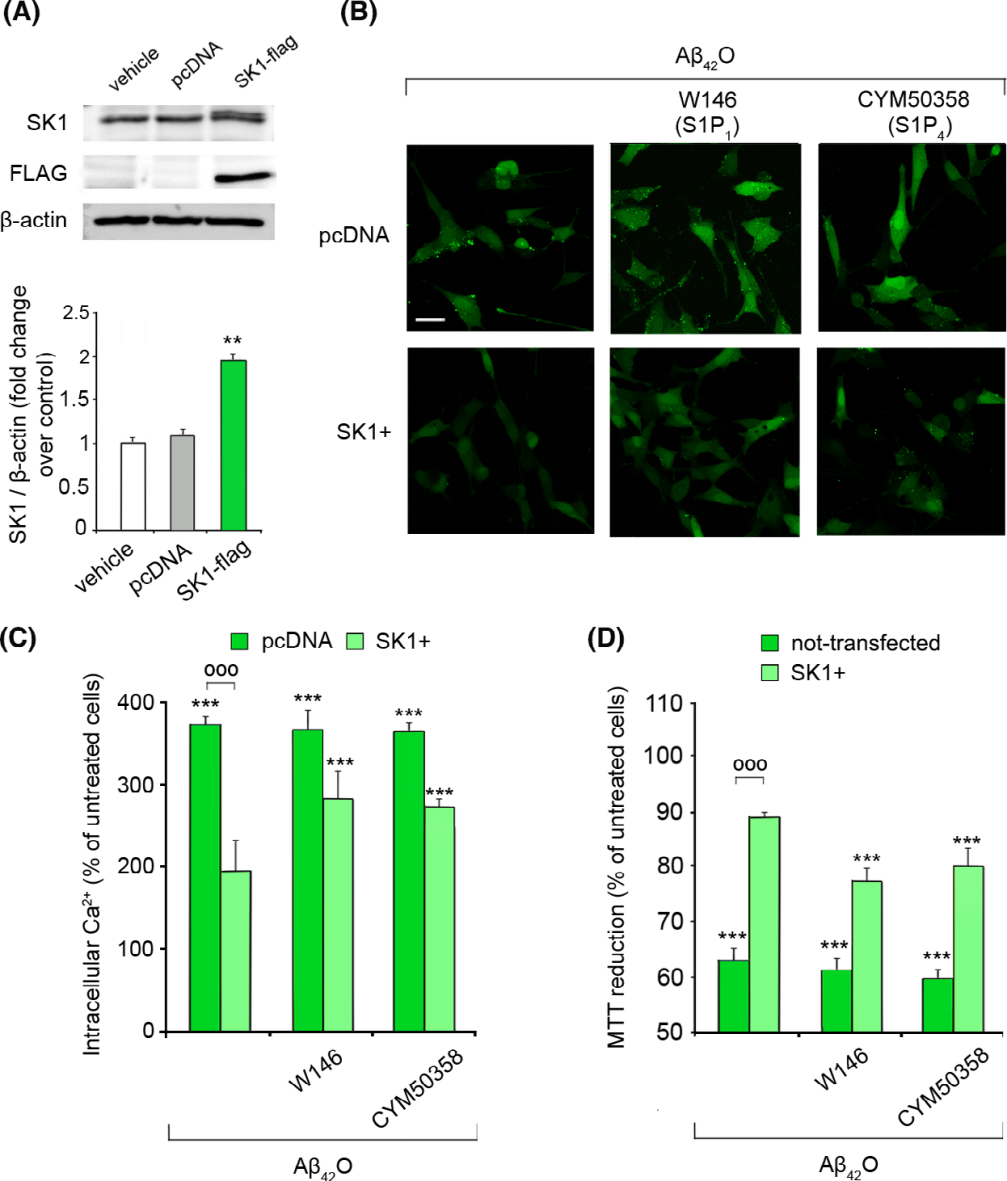
(A) Representative western blotting analysis of SK1‐flag in SH‐SY5Y cells. Cells were transiently transfected with pcDNA3‐human SK1‐flag plasmid or pcDNA3 empty vector (pcDNA), or only with the transfection reagent (vehicle). The histogram shows the band intensities, quantified by densitometric analysis and normalized to the expression of β‐actin. Data are expressed as fold increase relative to cells transfected with vehicle, set as 1. (B) Representative confocal microscope images showing the Ca^2+^‐derived fluorescence in SH‐SY5Y cells pre‐transfected with pcDNA or SK1‐Flag plasmid (SK1+), and then treated for 15 min with 3 μm Aβ_42_O, following 30 min of pre‐treatment with the S1PR antagonists W146 (S1P_1_) at 10 μm and CYM50358 (S1P_4_) at 1 μm. Cells were then loaded with the Fluo‐4 AM probe. (C) Semi‐quantitative analysis of the intracellular Ca^2+^‐derived fluorescence referring to panel (B). Data are expressed as the percentage of the value for untreated cells pre‐transfected with the respective plasmid. (D) MTT reduction in not‐transfected SH‐SY5Y cells, or pre‐transfected with SK1‐Flag plasmid (SK1+), and then treated for 24 h with 3 μm Aβ_42_O following 30 min of pre‐treatment with the S1PR antagonists W146 at 10 μm and CYM50358 at 1 μm. Data are expressed as the percentage of the value for untreated not‐transfected cells (vivid green), or untreated cells pre‐transfected with pcDNA (pale green). Experimental errors are SEM (n = 3). 15 μg of cell lysates (A), 80–120 cells (B, C) and 200 000–250 000 cells (D) were analysed per condition. Samples were analysed by Student's *t* test relative to untreated cells in panel (A) (**P < 0.01), or by one‐way ANOVA followed by Bonferroni's multiple‐comparison test relative to untreated cells (**P < 0.01, ***P < 0.001), and to pcDNA transfected or not‐transfected cells with the same treatment (°°°*P* < 0.001) in panels (C) and (D). Scale bars, 30 μm.

To investigate whether the sustained endogenous S1P production could overcome the Aβ_42_O detrimental effect, we transiently overexpressed Flag‐tagged SK1 in SH‐SY5Y (SK1+) cells, since SK1 is the isoform responsible for the generation of the S1P pool involved in the “inside‐out signalling” [25]. SK1+ cells displayed a significant overexpression of SK1 protein with respect to cells transfected with either control empty vector (pcDNA) or lipofectamine (vehicle), as shown using western blotting analysis (Fig. 9A). The intracellular Ca^2+^ increase induced by 3 μm Aβ_42_O was significantly lower in SK1+ cells (195 ± 35%) than in cells transfected with the empty vector (372 ± 9%) (Fig. 9B,C), which is in good agreement with the above‐reported data. The blockade of the S1P signalling in SK1+ cells by S1P_1_ and S1P_4_ specific antagonists partially prevented the protective action of S1P (Fig. 9B,C). Notably, the MTT reduction test also revealed a very low and non‐significant toxic effect of Aβ_42_O in SK1+ cells (Fig. 9D). In contrast, blockade of S1P_1_ and, to a lesser extent, S1P_4_ evoked a significant reduction in neuronal viability after 24 h (by 23 ± 5% and 20 ± 6%, respectively) as compared to untreated cells transfected with pcDNA (Fig. 9D). Overall, these data indicate that endogenous S1P, generated intracellularly by SK1, exerts a beneficial effect mediated by autocrine/paracrine mechanisms in neuronal cells challenged with toxic Aβ_42_O.

## Discussion

In this study, we provide evidence on the ability of S1P to significantly prevent dysfunction in primary rat cortical neurons and human neuroblastoma cells by reducing dramatically the Ca^2+^ dyshomeostasis induced by toxic prefibrillar aggregates of Aβ_42_. Oligomeric assemblies of Aβ_42_ are presently considered to be the key toxic agents responsible for neurodegeneration in AD [3, 5, 41]. Among the various oligomeric species so far characterized for Aβ_42_, the A11‐positive oligomers, also referred to as prefibrillar oligomers or A+, are most interesting because they have been shown to destabilize the lipid bilayer of cells [35, 54], cause dysfunction to neuroblastoma and pheochromocytoma cells [4, 8, 18, 35, 37] and be present in human AD brains [4, 38, 39].

These species, called here Aβ_42_O, were also previously reported to aberrantly interact with the neuronal membrane, thus destabilizing its integrity and evoking a massive entry of Ca^2+^ ions from the extracellular space [8, 11, 18, 37]. Our results indicate that S1P can protect neuronal cells challenged by Aβ_42_O from the abnormal increase in intracellular Ca^2+^ concentration and the following events in the toxicity cascade, such as mitochondrial dysfunction and caspase 3 activation. They also show that the protective effect of S1P was maximum at 100 nm, a physiological level that is responsible for the regulation of many intracellular processes triggered by Ca^2+^ signals [25]. Notably, S1P neuroprotection was mediated by S1P_1_ and S1P_4_ and, to a minor extent, also by S1P_3_ and S1P_5_, as demonstrated by the functional blockade of S1PR using both S1PR antagonists and by siRNA‐mediated S1PR silencing. Moreover, the protective effect of S1P was entirely lost in cells transfected with a combination of all siRNAs raised against the S1PR family and a synergistic effect was evident when the various S1PR agonists were co‐incubated. The outlined involvement of multiple S1PRs is in agreement with data reported in literature taking into consideration that S1PRs are co‐expressed in the same cell type, including neurons [55, 56], are coupled to multiple G proteins [55] and dictate multiple but also at times redundant intracellular downstream activated pathways. Actually, S1P_1_ exclusively couples to G_i_, S1P_3_ to G_i_, G_q_ and G_12_, and S1P_4_ and S1P_5_ couple to G_i_ and G_12_, respectively [44]. Among the plethora of intracellular signalling pathways evoked by S1P, our results indicate that the observed neuroprotective pathway is PTX sensitive, and therefore G_i_ dependent. These findings are in good agreement with the previously reported ability of the selective S1P_1_ agonist SEW2871 to reduce Aβ‐induced caspase‐3 activation, hippocampal neuronal death and cognitive impairment in AD rats [57]. In the same vein, more recently, it has been reported that the S1P analogue fingolimod rescues Aβ‐induced Ca^2+^ dyshomeostasis in hippocampal neurons through the activation of S1P_1_ and S1P_3_ [34]. Interestingly, the phosphorylated form of fingolimod was previously described as a potent neuroprotective agent against Aβ_42_O, able to stimulate the expression of brain‐derived neurotrophic factor (BDNF) in neurons [56]. This evidence was further confirmed in mice injected with Aβ_42_O, in which oral treatment with fingolimod restored the physiological levels of BDNF and, as a consequence, ameliorated memory impairment [58]. Importantly, the neuroprotective effect of S1P_4_, which decreases the neuronal Ca^2+^ overload induced by Aβ_42_O has been here highlighted for the first time. In this work, we also emphasize the role of S1P_5_ in the neuroprotective action of S1P. This is in line with the previously reported evidence on the beneficial effect of the S1P_5_ agonist A‐971432 in a non‐clinical animal model for AD [59].

Several reports have shown that Ca^2+^ influx can occur through a generic perturbation of the lipid bilayer and by the alteration of the activity of a variety of channels, such as the ionotropic receptors NMDARs [11, 12, 16, 18, 36, 60], with a prominent role played by the GluN2B subunit of these channels [19]. Here, we provide mechanistic details of the S1P_1_‐ and S1P_4_‐ and, to a lesser extent, S1P_3_‐ and S1P_5_‐mediated neuroprotective action of S1P on Aβ_42_O‐induced Ca^2+^ dysregulation. Our results show that GluN2B subunits of extrasynaptic NMDARs are internalized through a dynamin‐dependent endocytic process triggered by S1P signalling cascade, mainly occurring upon the activation of S1P_1_ and S1P_4_. A very similar effect has been observed in hippocampal neurons, where fingolimod evoked a very rapid relocation of such subunits at synaptic terminals, where their activation is not neurotoxic, but linked to pro‐survival actions through activation of long‐term potentiation [34]. Phosphorylated fingolimod was reported to induce a significant membrane accumulation of the GluN2B subunit in rat hippocampal slices upon prolonged S1P_1_ stimulation, associated with the activation of synaptic versus extrasynaptic NMDARs [61]. Collectively, these findings support a dual role for S1P signalling axis in modulating NMDARs: the first one in driving the rapid endocytic dynamin‐dependent internalization of extrasynaptic GluN2B subunit, and the second in stimulating their subsequent exposure at synaptic terminals, where such subunits are directly involved in the stimulation of neuroprotective pathways, leading to an increase in BDNF expression [62]. These conclusions are also in line with the general proposition that the same NMDAR subunit can activate both neuroprotective and neurotoxic cascades, in dependence on its subcellular localization in the context of synapses.

Fingolimod was also described for its ability to prevent the neurotoxicity of NMDA through the activation of S1P_1_ [63]. Interestingly, such molecule was reported to improve passive avoidance memory retrieval in relevant AD models as well as mem, one of the few available drugs for the symptomatic treatment of mild and severe AD [33]. Accordingly, in this work, we demonstrated the ability of S1P to rescue the abnormal calcium entry evoked by NMDA through the activation of S1P_1_, S1P_3_ and S1P_4_ to a very similar extent of mem in the presence of Aβ_42_O.

Binding of S1P or agonists, such as phospho‐fingolimod, to S1P_1_ results in phosphorylation of its serine‐rich C terminus and subsequent internalization via β‐arrestin‐mediated clathrin‐coated vesicles. After being internalized, the receptor can be consequently recycled back to the membrane, although binding of phospho‐fingolimod to S1P_1_ has been shown to induce its polyubiquitination and degradation [64]. Our results, showing that GluN2B subunits are internalized through an endocytic process triggered by S1P signalling mainly via activation of S1P_1_ and S1P_4_ and, to a minor extent, S1P_3_ and S1P_5_, reinforce the great protective value of S1P against the abnormal activation of extrasynaptic NMDARs in the cytotoxic cascade responsible for neurodegeneration in AD. These findings point the way for future research characterizing the process at the molecular level.

We also provided evidence on the crucial role played by SK1 in promoting Ca^2+^ homeostasis and neuronal survival mediated by S1P against Aβ_42_O‐induced neurotoxicity. Indeed, a significant down‐regulation of SK1 and SK2 expression was observed in the presence of Aβ_42_O, limiting S1P production and, consequently, its protective effect. Nevertheless, the mRNA expression levels of SPL as well as SPP1 were diminished, suggesting a reduced degradation of S1P over time, although previously reported data support the evidence that the enzymes involved in S1P biosynthesis, and not those responsible for its catabolism, are mainly responsible for the regulation of S1P cellular levels [65]. We also observed that Aβ_42_O induced a significant decrease in Spns2 expression, further reducing the pro‐survival effects of S1PR in neuronal cells. Our data indicate that in neuronal cells challenged by toxic Aβ_42_O, endogenous S1P generated intracellularly by SK1 exerts a beneficial effect mediated by autocrine/paracrine mechanisms after its extracellular export via the Spns2, according to previous studies in mouse myoblasts [53, 66]. Notably, the deleterious intracellular increase in Ca^2+^ levels and the mitochondrial dysfunction induced by Aβ_42_O were significantly prevented in SK1 overexpressing cells. Our data are in good agreement with previous evidence on the ability of the 25–35 fragment of Aβ to evoke a massive SK1 inactivation and with the finding that SK1 overexpression significantly prevented Aβ‐induced neurotoxicity in neuroblastoma cells [67].

In conclusion, our data provide new important insight into the neuroprotective role of S1P against toxic Aβ_42_ prefibrillar aggregates, both when added exogenously or when its endogenous formation is favoured. The present results indicate that S1P, binding to its receptors S1P_1_, S1P_3_, S1P_4_ and S1P_5_ stimulates the endocytic internalization of the GluN2B‐containing NMDARs, thus contributing to preventing the Ca^2+^ influx induced by deleterious Aβ_42_ oligomers. Taken together, our data point at S1P, S1PR agonists and SK1 activators as potential agents with therapeutic value for AD.

## Materials and methods

### Preparation of Aβ_42_O

Aβ_42_O were prepared as previously reported [35]. Briefly, the lyophilized peptide (Bachem, Bubendorf, Switzerland) was dissolved in 100% hexafluoro‐2‐isopropanol to 1 mm. The solvent was then evaporated under nitrogen and Aβ_42_ was resuspended in 50 mm NaOH at 1 mm, and then diluted in PBS to a final concentration of 25 μm. Then, the sample was centrifuged at 22 000 ***g*** for 30 min, the pellet was discarded, and the supernatant was incubated at 25 °C without agitation for 24 h to obtain Aβ_42_O. Aβ_42_ A‐ oligomers were prepared with the same procedure, at a final Aβ_42_ concentration of 25 μm, after incubation for 4 days [35]. The preparation of Aβ_42_ fibrils was assessed by dissolving the peptide in 50 mm NaOH at 1 mm, and then diluting it in PBS at a final concentration of 50 μm. The sample was then incubated at 25 °C for 24 h under quiescent conditions [35].

All oligomers and fibrils concentrations are reported as monomer equivalents.

### Cell culture

Authenticated human SH‐SY5Y neuroblastoma cells were purchased from A.T.C.C. (Manassas, VA, USA) and cultured in Dulbecco's modified Eagle's medium (DMEM), F‐12 Ham with 25 mm 4‐(2‐hydroxyethyl) piperazine‐1‐ethanesulfonic acid (HEPES) and NaHCO_3_ (1 : 1) supplemented with 10% FBS, 1 mm glutamine and 1% penicillin and streptomycin solution (Sigma‐Aldrich, St. Louis, MO, USA). Cells were maintained in a 5% CO_2_ humidified atmosphere at 37 °C and grown until 80% confluence for a maximum of 20 passages, and routinely tested to ensure that they were free from mycoplasma contamination [68]. For all the experiments, FBS was reduced to 0.5%. Primary rat cortical neurons (Thermo Fisher Scientific, Waltham, MA, USA) were plated and maintained in Neurobasal medium (Thermo Fisher Scientific) supplemented with 0.5 mm GlutaMAX (Gibco, Thermo Fisher Scientific) and 2% (v/v) B‐27 serum‐free complement (Gibco, Thermo Fisher Scientific) at 37 °C in a 5% CO_2_ humidified atmosphere. Every 2 days, the medium was partially replaced with a fresh one and the experiments were performed 14 days after plating, as previously reported [18, 37, 69], reducing GlutaMAX and B‐27 serum‐free complement concentrations to 0.25 mm and 1% (v/v) respectively.

### RNA interference

SH‐SY5Y cells seeded on glass coverslips were transfected with 25 nm Stealth RNAi, referred to as control siRNA (Thermo Fisher Scientific), or with 25 nm siRNA against the GluN2B coding gene GRIN2B (Thermo Fisher Scientific), or with 25 nm siRNA against S1P_1_ (Merck Millipore, Burlington, MA, USA), S1P_2_ (Merck Millipore), S1P_3_ (Merck Millipore), S1P_4_ (Merck Millipore) and S1P_5_ (Merck Millipore), or with a combination of all the siRNAs raised against S1PRs, using Lipofectamine 3000 (Life Technologies, Carlsbad, CA, USA), according to the manufacturer's instructions, with 7 μL of lipofectamine and 10 μL of 5 mg·L^−1^ transferrin in DMEM for 3 h in a 5% CO_2_ humidified atmosphere at 37 °C. Then, the transfection medium was replaced with fresh complete culture medium, in which cells were incubated for 72 h.

### Cell transfection with SK1 plasmid

To obtain cells overexpressing wild‐type SK1, SH‐SY5Y were plated onto 60 mm dishes and transfected using a mix of pcDNA3‐human SK1‐Flag plasmid (kindly provided by S. Pitson) [70] or empty vector (pcDNA) and Lipofectamine 2000 reagent (1 mg·mL^−1^) (Life Technologies) as previously described [71]. After 36 h, cells were utilized for the experiments. Overexpression was checked by western blotting analysis employing anti‐Flag (Sigma‐Aldrich) and anti‐SK1 antibodies (ECM Biosciences, Versailles, KY, USA).

### Measurement of cytosolic Ca^2+^ levels

In a set of experiments, SH‐SY5Y cells plated on glass coverslips were incubated for 15 min with 1 μm ionomycin, or with 3 μm Aβ_42_ M, Aβ_42_ A‐, Aβ_42_O and fibrils. In another set of experiments, Aβ_42_O were added to the culture medium of SH‐SY5Y cells seeded on glass coverslips for 15 min at increasing concentrations (0.1, 0.3, 1, 3 and 10 μm). In one set of experiments, 3 μm Aβ_42_O were added to the culture medium of SH‐SY5Y cells for 5, 10 and 15 min, in the absence or presence of S1P (Calbiochem, San Diego, CA, USA) at 100 nm. In a separate set of experiments, SH‐SY5Y cells were pre‐treated with 200 ng·mL^−1^ PTX for 4 h and then treated with 3 μm Aβ_42_O in the absence or presence of 100 nm S1P. In another set of experiments, SH‐SY5Y cells were pre‐treated for 30 min with the selective S1PR antagonists W146 (Avanti Polar Lipids, Alabaster, AL, USA) at 10 μm (S1P_1_), JTE013 (Avanti Polar Lipids) at 1 μm (S1P_2_), CAY10444 (Cayman Chemical, Ann Arbor, MI, USA) at 5 μm (S1P_3_), CYM50358 (kindly gifted by E. Roberts) [72] at 1 μm (S1P_4_) and VPC23019 (Avanti Polar Lipids) at 1 μm (S1P_1/3_); cells were then treated with Aβ_42_O at 3 μm for 15 min, in the absence or presence of 100 nm S1P. SH‐SY5Y cells were also treated for 15 min with 3 μm Aβ_42_O in the presence of the selective S1PR agonists SEW2871 (Calbiochem) at 10 μm (S1P_1_) and CYM50308 (kindly gifted by E. Roberts) at 1 μm (S1P_4_), respectively. In a separate set of experiments, SH‐SY5Y cells were transfected with control siRNA, or with siRNAs against S1P_1_, S1P_2_, S1P_3_, S1P_4_ and S1P_5_ and with a combination of all the siRNAs raised against S1PRs as reported in the previous section, and then treated for 15 min with 3 μm Aβ_42_O in the absence or presence of 100 nm S1P. In a set of experiments, SH‐SY5Y cells were treated with 3 μm Aβ_42_O for 15 min, in the absence or presence of a 60 min pre‐treatment with 10 μm mem (Sigma Aldrich), or after transfection with control siRNA or with siRNA against GluN2B. In a separate set of experiments, primary rat cortical neurons were treated with 3 μm Aβ_42_O for 15 min, in the absence or presence of 100 nm S1P, or with NMDA (Sigma Aldrich) at 1 mm for 15 min, in the absence or presence of 100 nm S1P, and with or without a pre‐incubation of 30 min with the selective S1PR antagonists W146 at 10 μm (S1P_1_), CAY10444 at 5 μm (S1P_3_), or CYM50358 at 1 μm (S1P_4_). In another set of experiments, SH‐SY5Y cells were pre‐incubated for 30 min with 50 μm dyn (Sigma Aldrich), and then treated with 3 μm Aβ_42_O and 100 nm S1P for 15 min. In a set of experiments, SH‐SY5Y cells pre‐transfected with pcDNA or with SK1‐Flag plasmid were treated for 15 min with 3 μm Aβ_42_O, in the absence or presence of a 30 min pre‐incubation with the selective S1PR antagonists W146 at 10 μm (S1P_1_), and CYM50358 at 1 μm (S1P_4_).

Cells were then loaded with 4.5 μm fluo‐4 AM (Thermo Fisher Scientific) for 10 min and the cytosolic Ca^2+^ levels were detected after excitation at 488 nm by a TCS SP8 scanning confocal microscopy system (Leica Microsystems, Mannheim, Germany), equipped with an argon laser source. A series of 1‐μm‐thick optical sections (1024 × 1024 pixels) was taken through the cell depth for each sample using a Leica Plan Apo 63× oil immersion objective, and all sections were projected as a single composite image by superimposition. The confocal microscope was set at optimal acquisition conditions, e.g. pinhole diameters, detector gain and laser powers. Settings were maintained constant for each analysis. Images were then analysed using the imagej (NIH, Bethesda, MD, USA) software (Rasband 1997–2018). The fluorescence intensities were typically expressed as the percentage of that measured in untreated cells.

### MTT reduction inhibition assay

The mitochondrial status of SH‐SY5Y cells seeded in 96‐well plates was evaluated by the 3‐(4,5‐dimethylthiazol‐2‐yl)‐2,5‐diphenyltetrazolium bromide (MTT) assay, as reported previously [73]. In a set of experiments, increasing concentrations (0.1, 0.3, 1, 3 and 10 μm) of Aβ_42_O were added for 24 h to the culture medium of SH‐SY5Y cells seeded on 96‐well plates. In another set of experiments, SH‐SY5Y cells were treated for 24 h with Aβ_42_O at 3 μm in the absence or presence of increasing concentrations (10, 30, 100, 300 nm and 1 μm) of S1P. In the same set of experiments, SH‐SY5Y cells were treated for 24 h with Aβ_42_O at 3 μm following a 15 min pre‐incubation with 100 nm S1P. Increasing concentrations (100, 300 nm and 1 μm) of S1P were also tested as a control. In a separate set of experiments, SH‐SY5Y cells were treated for 24 h with Aβ_42_O at 3 μm in the absence or presence of 100 nm S1P, with or without a 30 min pre‐treatment with the selective S1PR antagonists W146 at 10 μm(S1P_1_), CAY10444 at 5 μm (S1P_3_) and CYM50358 at 1 μm (S1P_4_), and with a combination of all the S1PRs antagonists at the previously reported concentrations. In another set of experiments, SH‐SY5Y were transfected with pcDNA or with the SK1‐Flag plasmid, and then treated for 24 h with Aβ_42_O at 3 μm in the absence or presence of a pre‐treatment with the selective S1PR antagonists W146 at 10 μm (S1P_1_) and CYM50358 at 1 μm (S1P_4_).

After treatment, the culture medium was removed, and the MTT solution was added to the cells for 4 h. The formazan product was then solubilized with cell lysis buffer (20% sodium dodecyl sulfate and 50% *N*,*N*‐dimethylformamide, pH 4.7) for 1 h. The absorbance values of blue formazan were determined at 590 nm with the microplate manager® Software (Bio‐Rad, Hercules, CA, USA). Cell viability was typically expressed as the percentage of MTT reduction in treated cells as compared to the untreated ones, unless otherwise indicated.

### Caspase‐3 activity assay

SH‐SY5Y cells plated on six‐well plates were treated for 24 h with Aβ_42_O at 3 μm in the absence or presence of 100 nm S1P; 100 nm S1P alone was also tested as a control. After treatment, cells were washed twice with PBS and lysed for 20 min at 4 °C in 20 mm Tris–HCl buffer (pH 7.4) containing 250 mm NaCl, 2 mm EDTA, 0.1% Triton X‐100, 5 μg·mL^−1^ aprotinin, 5 μg·mL^−1^ leupeptin, 0.5 mm phenylmethylsulfonylfluoride, 4 mm sodium vanadate and 1 mm DTT. The lysis was completed by sonication, and total protein content was determined in the clarified lysates with the Bradford reagent. Aliquots of total proteins were diluted in 50 mm HEPES‐KOH buffer (pH 7.0) containing 10% glycerol, 0.1% 3‐[(3‐cholamidopropyl)‐dimethylammonio]‐1‐propane sulfonate, 2.0 mm EDTA and 10 mm DTT. Caspase‐3 activity was determined, as described in [74, 75], by incubating a protein sample for 2 h at 37 °C in the presence of 50 μm Ac‐DEVD‐AFC (fluorimetric substrate; excitation 405 nm and emission 505 nm). To determine non‐specific substrate degradation, the assays were also performed by pre‐incubating total protein samples for 15 min at 37 °C with or without the specific caspase inhibitor (200 nm Ac‐DEVD‐CHO) before substrate addition.

### Immunostaining of GluN2B subunit of NMDARs

SH‐SY5Y seeded on glass coverslips cells were transfected with control siRNA, or with siRNA against S1P_1_, S1P_2_, S1P_3_, S1P_4_, S1P_5_ and with a combination of all the siRNAs raised against S1PRs as reported previously, and then treated for 15 min with S1P at 100 nm, or with Aβ_42_O at 3 μm in the absence or presence of S1P 100 nm. In the same set of experiments, SH‐SY5Y cells were also pre‐treated for 30 min with 50 μm dyn. Cells were then fixed with 2% (v/v) paraformaldehyde. After washing twice with PBS, the GluN2B subunit was detected with 1 : 400 diluted anti‐NMDAε2 antibody (Santa Cruz Biotechnology, Dallas, TX, USA), and subsequently with 1 : 1000 diluted Alexa Fluor 633‐conjugated anti‐mouse secondary antibodies (Thermo Fisher Scientific). To detect only GluN2B exposed on the cell surface, the plasma membrane was not permeabilized, thus preventing antibody internalization. Fluorescence emission was detected after excitation at 633 nm by the TCS SP8 scanning confocal microscopy system described above. GluN2B‐derived fluorescent puncta were counted using imagej software after subtracting the background and setting constant thresholds for all the analysed images.

### Quantitative real‐time reverse transcription PCR

SH‐SY5Y cells were plated on six‐well plates and then treated for 24 h with Aβ_42_O at 3 μm. Total RNA from SH‐SY5Y cells was then extracted using TRI Reagent® (Sigma‐Aldrich). Then, 1–2 μg of RNA was reverse transcribed using the high‐capacity cDNA reverse transcription kit (Applied Biosystems, Foster City, CA, USA). TaqMan gene expression assays were used to perform real‐time PCR to quantify the mRNA expression of S1P metabolism enzymes (SK1 and SK2), and S1P‐specific transporter Spns2. Each measurement was carried out in triplicate using the CFX96 Touch™ Real‐Time PCR Detection System (Bio‐Rad) as described previously [75, 76], by simultaneous amplification of the target sequence together with the housekeeping gene β‐actin. The $2^{-\Delta \Delta Ct}$ method was applied as a comparative method of quantification [77], and data were normalized to β‐actin expression.

### Western blotting analysis

In a set of experiments, increasing concentrations (0.1, 0.3, 1 and 3 μm) of Aβ_42_O were added for 24 h to the culture medium of SH‐SY5Y cells seeded on six‐well plates. In another set of experiments, SH‐SY5Y cells were plated on six‐well plates and then treated for 24 h with Aβ_42_O at 3 μm. Cells were then collected and lysed 30 min at 4 °C in a buffer containing 50 mm Tris, pH 7.5, 120 mm NaCl, 1 mm EDTA, 6 mm EGTA, 15 mm Na4P_2_O_7_, 20 mm NaF, 1% Nonidet and protease inhibitor cocktail. Then, lysates centrifuged at 10 000 ***g***, 15 min at 4 °C and 15 μg of protein from total cell lysates were used to perform SDS/PAGE and western blotting analysis in order to evaluate with specific antibodies caspase 3 cleavage (Cell Signalling) or expression of SK1 (ECM Biosciences), SK2 (ECM Biosciences), Spns2 (kindly gifted by T. Nishi). PDVF membranes were incubated overnight with the primary antibodies at 4 °C and then with specific secondary antibodies for 1 h at room temperature. The binding of the antibodies with the specific proteins has been detected by chemiluminescence employing Amersham Imager 600 (GE Heathcare, Buckinghamshire, UK). Densitometric analysis was performed by imagej software.

### Statistical analysis

All data were presented as means ± standard error of mean (SEM). Comparisons between the different groups were performed by Student's *t* test or one‐way ANOVA followed by Bonferroni's post‐comparison test, or *post hoc* test, by using graphpad prism 7.0 software (San Diego, CA, USA) .

## Conflict of interest

The authors declare no conflict of interest.

## Author contributions

AB conceptualized the study, developed methodology, investigated and visualized the study, wrote the original draft, and wrote, reviewed and edited the manuscript. RC developed methodology, investigated the study, wrote the original draft, wrote, reviewed and edited the manuscript, and acquired funding. GF investigated and visualized the study. CB investigated and visualized the study, and wrote, reviewed and edited the manuscript. FCe, PB and FCh conceptualized the study, wrote, reviewed and edited the manuscript, and acquired funding. CD conceptualized the study, wrote the original draft, wrote, reviewed and edited the manuscript, administrated the project and acquired funding. CC conceptualized the study, wrote the original draft, wrote, reviewed and edited the manuscript, supervised the study, administrated the project and acquired funding.

### Peer review

The peer review history for this article is available at https://publons.com/publon/10.1111/febs.16579.

## Data Availability

The authors confirm that all data needed to evaluate the conclusions of this study are available within the article.
